# Challenges of Managing Animals in Disasters in the U.S.

**DOI:** 10.3390/ani5020173

**Published:** 2015-03-25

**Authors:** Sebastian E. Heath, Robert D. Linnabary

**Affiliations:** 1 Program Development and Analysis, Federal Emergency Management Agency (FEMA), Washington, DC 20472, USA; 2 Retired from College of Veterinary Medicine, University of Tennessee, Knoxville, TN 37996, USA; E-Mail: rlinnaba@comcast.net

**Keywords:** animals, disaster, emergency management, planning, preparedness, mitigation, response, recovery, pet overpopulation

## Abstract

**Simple Summary:**

This article describes common challenges to managing animals in disasters in the US, summarizes how some of these challenges are being met and makes recommendations on how to overcome others. Many predictable adverse situations affecting animals and their owners can be prevented when communities develop a comprehensive emergency management strategy that integrates animal care into planning, preparedness, mitigation, and recovery activities, as well as response.

**Abstract:**

Common to many of the repeated issues surrounding animals in disasters in the U.S. is a pre-existing weak animal health infrastructure that is under constant pressure resulting from pet overpopulation. Unless this root cause is addressed, communities remain vulnerable to similar issues with animals they and others have faced in past disasters. In the US the plight of animals in disasters is frequently viewed primarily as a response issue and frequently handled by groups that are not integrated with the affected community’s emergency management. In contrast, animals, their owners, and communities would greatly benefit from integrating animal issues into an overall emergency management strategy for the community. There is no other factor contributing as much to human evacuation failure in disasters that is under the control of emergency management when a threat is imminent as pet ownership. Emergency managers can take advantage of the bond people have with their animals to instill appropriate behavior amongst pet owners in disasters.

## 1. Introduction

In the last decade there have been many large-scale disasters that have raised awareness of the needs of animals in disasters [1], and there have been many more local, small-scale incidents that have passed without being given great attention. Stories of the plight of animals in disasters are common in the media and often described as being solely the result of the disaster. However, disasters rarely create new situations, in most cases, disasters simply expose underlying systemic vulnerabilities in a community by suddenly opposing chronically unmet needs with equally chronic insufficient resources. For example in the US we have seen how power grids fail as a result of demands for electric power increasing incrementally, but designs not having been kept current. Developments in picturesque floodplains continue to accommodate an increasing population growth, but remain susceptible to flooding. As bridges and other infrastructure age without constant upgrades to their engineering their threshold for failure is lowered. Similarly, the issues affecting animals during the response to disasters are rarely new, they are rather mostly exacerbations of pre-existing conditions. When the solutions to issues affecting animals in disasters are viewed through the lens of response, the management of animals is crisis-driven and temporary rather than directed at long-term and systemic goals by correcting underlying systemic deficiencies. A response driven focus has been a persistent challenge to managing animals in disasters in the U.S., which may explain why so many of the animal issues seen in disasters occur repeatedly. In this article, we will try to elucidate the connections between pre-existing conditions that give rise to typical issues seen during the response to disasters and give examples of how a comprehensive emergency management strategy could mitigate commonly occurring issues affecting animals in disasters.

Over the past 20 years the U.S. have developed many resources for the care of animals in disasters. However, despite much progress having been made since the authors’ earliest work on the subject [2,3], the recurrence of some of the issues affecting animals in disasters in the U.S. speaks volumes as to the underlying vulnerability many communities still face. In contrast, in the professional emergency management community there are many proven strategies to permanently reduce the adverse impacts of disasters on communities’ infrastructures. In this article we propose that applying the principles of emergency management to the care of animals in disasters leads to improvements of public and animal health that are sustainable and will likely reduce the incidence of animal issues arising in disasters.

Emergency managers recognize five phases of the emergency management lifecycle:

- Planning,
- Preparedness,
- Mitigation,
- Response,
- Recovery.

Through an understanding of what each of these phases entails we intend to guide the reader to gain insight into the value of applying a comprehensive emergency management strategy to the care of animals in disasters.

## 2. Planning

Planning impacts all phases of disaster. Well thought out and implemented plans incorporate the needs of animals and their owners into the Emergency Operations Plan of the community, identify and prioritize realistic threats, hazards, and vulnerabilities, define the response mission and goals, realistically describe existing capabilities to meet the response goals, conduct capability gap analyses between actual and desired capability, and develop business plans to close any capability gaps. Planning is critical because capability gaps can occur and be filled in any phase of disaster, and because the process of planning itself is a critical part of creating a functional emergency management system. During an active planning process, stakeholders have the opportunity to work side by side on problem solving and get to know each other and the community long before disaster strikes and in ways that are beneficial to the response to a disaster. When planning and training has been inadequate, parties responsible for the response and recovery often meet each other during the response. Under such circumstances they depend on one another to make decisions over human lives, high-in-demand resources, including large sums of money, often under cramped and extremely stressful conditions without knowing each other well. Clearly trying to solve problems under such circumstances is not conducive to effectively manage a community’s systemic animal-related needs. Effective planning can prevent this type of *ad hoc* emergency management.

### 2.1. Planning Improves Mitigation

Examples of planning efforts for animals include identifying the need for stronger legislation, establishing a legislative action group, and creating a stronger commitment to make resources available for the care of animals by assigning roles and responsibilities of responders and by delegating authority. Other local planning efforts that support mitigation measures are those that affect resource allocations including commitments of human shelter managers to allow the co-location of people and animals during disasters. For shelters to operate effectively, planning efforts can identify appropriate policies for management of animals, including under what conditions shelters will become available, where animals will be housed, who carries which liabilities, staffing levels and training of appropriate animal care personnel, such as animal control officers, animal technicians, veterinarians, and volunteers, and what responsibilities managers have, as well as any waivers that owners may be required to sign. Best practices for animal shelter policy/guidelines have been published by the National Alliance of State Animal and Agricultural Emergency Programs (NASAAEP) [4]. In addition we recommend that animal owners wanting to care for animals be limited to general care of their own animals, and for shelters to establish procedures, such as a requirement for a family member to remain in the shelter as long as the animal is housed there or for the owner to agree to a release of ownership for the animal in case they leave the shelter without the animal and do not return.

### 2.2. Planning Improves Preparedness

Planning can result in better preparedness by identifying the need for training and exercises that build response capability and by developing a business plan on how to fund, conduct, and evaluate the impact of training, exercises, and real world events. Particular emphasis during planning should be placed on identifying special needs groups with animals, including the elderly who may need additional resources to evacuate with their pets, service animals that may have owners with special needs, and the ubiquitous owner with too many pets to care for on their own. Specialized response animals, such as Canine Urban Search and Rescue dogs may need to have designated locations identified in advance for recuperation between shifts during the response. Along these lines, the National Fire Protection Association (NFPA) has drafted a “Standard for Mass Evacuation and Sheltering” which includes an annex on “Service Animals and Pets”. Once this standard has been approved (slated for implementation in 2017) it will become mainstream practice for emergency management and fire departments across the U.S. to plan for the care of animals in disasters in the U.S. [5]. 

### 2.3. Planning Improves Response and Recovery

Examples of planning to improve response include pre-identifying housing and other resources that may be needed during a disaster and that will allow animals to be cared for. And last but not least, planning for the recovery from disasters should aim to define the desired future state of the community and should engage the community at large, community planners, developers, financiers, and subject matter experts. For many, planning for the recovery from a disaster is a variation on project risk management for ongoing community development. In other words community development should assess the impact of how a potential disaster could impair planned progress, including grasping the opportunity to reprioritize the direction of a community’s development.

## 3. Preparedness

Preparedness involves training, exercising, and credentialing responders, as well as creating public awareness that empowers individuals to care for themselves and their dependents.

There is tremendous enthusiasm in the U.S. to prepare for the care of animals in disaster. However, much of the preparedness activities are conducted by groups and individuals who are not integrated within the community’s official emergency management. When this happens, local volunteers become frustrated because they are not called in to help or, or worse, they self-deploy and operate outside of the official response. Later we will illustrate why that approach might be good for fundraising, but, because this is a response-centric approach, an independent response does little to address underlying problems that led to the need to respond in the first place. Working outside of the official channels of emergency management is generally counterproductive when trying to build a sustainable capacity to manage animals in disasters.

In an attempt to build an emergency management capacity in the U.S., the Federal Emergency Management Agency (FEMA) has established a credentialing system called the National Incident Management System (NIMS) that defines the minimum qualifications for most response positions within the Incident Command System (ICS). A subset of these responders are the Animal Emergency Responders (AER), who, once credentialed, can assume official positions in emergency management and, therefore, give credibility to issues surrounding animals and their owners in disasters. The basis for a national system for credentialing emergency responders is to establish common performance standards for responders as well as common definitions for resources, *i.e.*, credentialing is resource typing of response personnel. The official AER credentials are summarized in Table 1 [6]. At the time of publication of this article the team credentials were still under review. Draft copies of these can be obtained from the authors.

**Table 1:** Criteria used to credential Animal Emergency Responders (AER) [6].

Credential	Criteria
**Education**	Formal instruction based upon a curriculum that prepares an individual with the core knowledge and skill for entry into a discipline and for performing a job title
**Training**	Instruction and activities that enhance and an individual’s core knowledge, increase skill set and proficiency, and strengthen and augment abilities
**Experience**	Time required functioning in a job title for an individual to attain proficiency in applying knowledge, skills, and abilities
**Physical/Medical Fitness**	Physical and medical considerations that, when applied, help to ensure safe performance in risky environments
**Certification**	Designation granted by Authority Having Jurisdiction that an individual has met the requirements and achieved specific knowledge, skills, and abilities
**Licensing**	Legal designation granted by Authority Having Jurisdiction, indicating that a person has met the necessary legal requirements to function in a job title. Licensing requirements are established by a State or federal agency to permit persons to practice their trade or profession
Because the needs of animals are highly specialized, each animal emergency responder is further typed based on his/her qualifications, experience or expertise of dealing with certain species. Hence credentialed AERs also meet species pre-requisites for one or more of the following groups: Companion animals (such as dogs, cats and household pets); Equines; Livestock (such as cattle, sheep, goats, pigs); Avian; Zoo, non-domestic, and exotic species (grouped together as “Non-domesticated”).	

FEMA has also developed several animal specific training courses. These are the Independent Study courses Animals in Disasters (IS-10) [7], Community Preparedness (IS-11) [8], and Livestock in Disasters (IS-111) [9]. These, together with basic training in Incident Command (ICS-100, ICS-200) [10,11] and expertise of handling animals form the basis of the credentialing system for entry-level animal emergency responders. FEMA’s Emergency Management Institute provides many other training opportunities for dedicated responders to become credentialed in other positions [12].

Although not specific to the care of animals, because of the huge outpouring of help for the care of animals in disasters by well meaning, but non-credentialed, responders, the authors recommend that as part of a community’s preparedness activities a credentialed Volunteer Manager be identified ahead of time to be included in the animal emergency response teams [13]. A volunteer manager should set expectations for volunteers, assign duties that non-credentialed personnel can perform safely, such as food preparation, feeding and exercising animals, comforting non-aggressive pets, establish duty schedules, and ensure that the work environment is as safe as can be under the circumstances. Because of the rarity of large scale events in any one location, some communities have developed procedures to go into effect during a disasters, such as an onsite and as-needed registration for all volunteers, including animal care personnel, as part of their preparedness activities.

In addition to having developed a framework for training, FEMA provides grants to States and local communities that can be used to pay for conducting and attending training and exercises, as well as backfilling positions of personnel attending training and exercises. Although copious funding is available for preparedness, real world experience has shown that without established authorities (*i.e.*, a lack of adequate mitigation) and funding for a response, even qualified responders may not be able to be compensated for deploying to a real event. This became evident during the 2009 influenza pandemic that swept the U.S., when many States and the federal government realized that there were insufficient authorities to declare and pay for the State or federal response to a public health emergency. Even though funding was available to cover the costs of training and exercises for the response to an influenza epidemic, including overtime and backfill costs, these could not be used during an actual response. Many of these shortfalls were addressed in the subsequently enacted Pandemic and All Hazards Preparedness Act that (PAHPA) Title III Section 302, which is good example of real world event leading to intense planning and successful mitigation [14].

## 4. Mitigation

Mitigation is the permanent alteration of the physical, legal, financial, and other resource environments to reduce the adverse impacts of disasters.

Classic examples of mitigation include instituting appropriate building codes for known hazards, such as earthquakes and floods, enforcing transportation routes for hazardous materials to circumvent human population centers, and acquiring specialized equipment needed in the event of a disaster. Mitigation efforts that reduce adverse impacts on the animal health infrastructure, *i.e.*, the public, private and non-government services, and facilities in a community that support animal wellbeing, include legislation, regulations, and their enforcement as well as other commitments that set minimal standards for the care of animals and make available resources to achieve and maintain those standards during disasters. Protection from extreme weather, such as wind breaks for livestock exposed to blizzards, elevated dirt mounds for livestock living in floodplains to move to as flood waters rise, and ensuring that sufficient and appropriate feed and water are available to animals exposed to temperature extremes are examples of changes to the physical environment that mitigate disasters for livestock. Insurance is another mitigation tool and, although not available in the U.S., countrywide insurance plans have been developed in Peru [15] and Kenya [16] that disperse the financial risk resulting from animal disease outbreaks and droughts, respectively, to raising livestock during non-disaster times and ensuring adequate funding for response and recovery once disaster strikes.

Examples of occurrences where mitigation of the needs of animals in disasters have fallen short include the need for housing large numbers of stray animals, and external groups leading response efforts in spite of local authorities. As discussed later in the Response section of this article, most of these animals are most likely either stray or become strays at the time of the disaster. They become strays under similar circumstances when owners’ lives are disrupted, (e.g., by moving, foreclosure, divorce) and the owners leave their pets to fend for themselves or have them euthanized. If intact animals are “set free” they will contribute to the breeding pool of stray animals leading to a huge problem of overpopulation in the U.S. In the U.S. it has been estimated that 1.2–1.4 million unwanted cats and dogs are euthanized every year [17]. To what extent this phenomenon occurs outside of the U.S. is unclear, but to manage this issue effectively in the U.S., we should regrettably, and much to the contempt of many response-centric managers and animal advocates, assume that animals found at disaster sites without an identifiable owner are strays and cohorts of the pet overpopulation problem in the U.S. This assumption is necessary to get to the core of recurring problems and can only be solved in the long-term if common underlying causes are identified and corrected. Until communities address pet overpopulation it is likely that large numbers of stray animals will be a recurring issue in large-scale disasters. This is an example of how a pre-existing conditions emerge as a crisis when disaster strikes; the pre-existing condition being the mismatch of chronic excess of unwanted animal populations (demand) and limited capabilities of communities’ animal health infrastructure (resource). In the following are some examples of mitigation measures that have contributed to reducing the negative impacts of disasters on animals and by addressing animal population control in non-disaster times.

**Figure 1:**
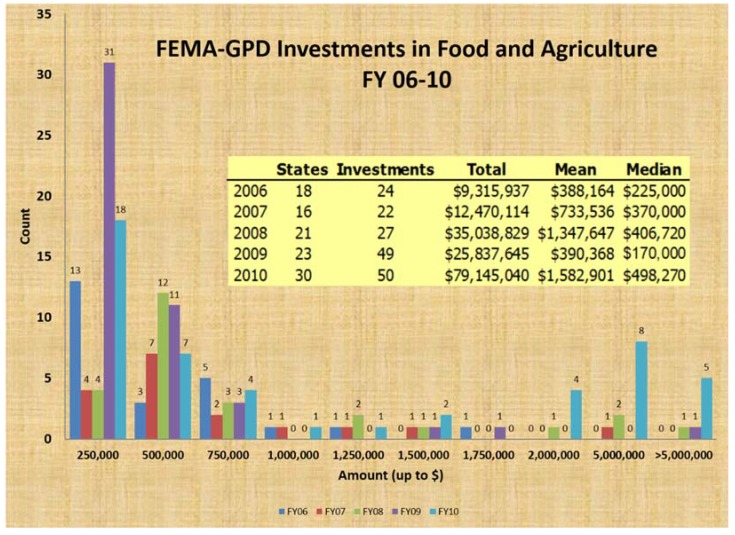
Summary of the number of States and projects funded to support disaster preparedness for animals and the approximate amount of funding per project (Fiscal Years 2006–2010); most projects supported animal agriculture. Source: FEMA, 2011.

**Figure 2:**
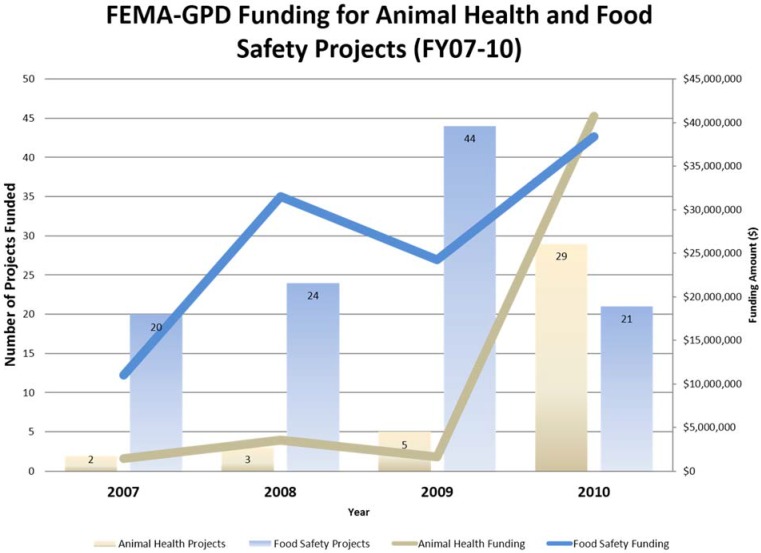
Summary of the total number of projects funded and funding obligated by FEMA to support disaster preparedness for animals (Fiscal Years 2007–2010); most projects supported animal agriculture. Source: FEMA, 2011.

At the national level, in the U.S., an example of effective legislation is the Pet Evacuation and Transportation Standards (PETS) Act, which addresses “the needs of individuals with pets and service animals prior to, during, and following a major disaster or emergency” by making the Federal Emergency Management Agency (FEMA) preparedness grant funds available “to the States and local authorities for animal emergency preparedness purposes, including the procurement, construction, leasing, or renovating of emergency shelter facilities and materials that will accommodate people with pets and service animals” [18]. Coincidental with the passing of this law and the recovery from Hurricane Katrina (in 2005) many States have greatly increased the use of federal FEMA preparedness grant funding for activities involving animals; notably, however, many of these activities supported animal agriculture and not pets (Table 2 [19]; Figure 1 and Figure 2).

**Table 2:** Examples of the types of projects funded by the Federal Emergency Management Agency (FEMA) to advance disaster preparedness for animals since the enactment of the Pet Evacuation and Transportation Standards (PETS) Act; most projects supported animal agriculture [19].

Project Examples	
Establish/enhance agro-terrorism preparedness capabilities	Develop/enhance State and local geospatial data system/GIS
Develop/enhance plans, procedures, and protocols	Develop/enhance interoperable communications systems
Enhance capabilities to respond to all-hazards events	Establish/enhance fusion center
Establish/enhance mass care shelter and alternative medical facilities operations	Establish/enhance cyber security program
Establish/enhance regional response teams	Establish/enhance citizen/volunteer initiatives
Establish/enhance emergency plans and procedures to reflect the National Response Plan	Establish/enhance sustainable Homeland Security Planning Program
Develop/enhance homelandsecurity/emergency management organization and structure	Establish/enhance a terrorism intelligence/early warning system, center, or task force
Establish/enhance sustainable homeland security training program	Enhance capability to support economic and community recovery
Administer and manage the Homeland Security Grant Program	Establish/enhance public-private emergency preparedness program
Enhance integration of metropolitan area public health/medical and emergency management capabilities	Establish/enhance Citizen Corps Councils
Establish/enhance a public health surveillance system	Establish/enhance emergency operations center
Enhance capability to perform post-incident structural damage and mitigation assessment	Establish/enhance citizen awareness of emergency preparedness, prevention, and response measures
Assess vulnerability of and/or harden/protect critical infrastructure and key assets	Establish/enhance sustainable homeland security exercise program

At the State level, legislation can mitigate animal disasters by including laws that clearly define who is in charge and what resources are available to them. All States in the U.S. clearly define the office of the State Animal Health Official (usually the State Veterinarian) who has the lead on managing outbreaks of regulated animal diseases, such as Foot and Mouth Disease, Avian Influenza, and Classical Swine Fever. However, there is no uniformity in who has the authority to care for pets that are free of notifiable diseases in natural disasters. This is in part because most State animal welfare laws have often been written piecemeal over decades and, as a result, often do not have comprehensive objectives or set a uniform tone on expected standards of care or enforcement. In an attempt to offer an effective solution, Garvey *et al.* analyzed State legislation on animal care and drafted model legislations [20]. Based on the analysis of state laws, the authors recommend where mitigation efforts at the State level could be improved in the U.S. and offers an example of what comprehensive animal care legislation could look like.

**Figure 3:**
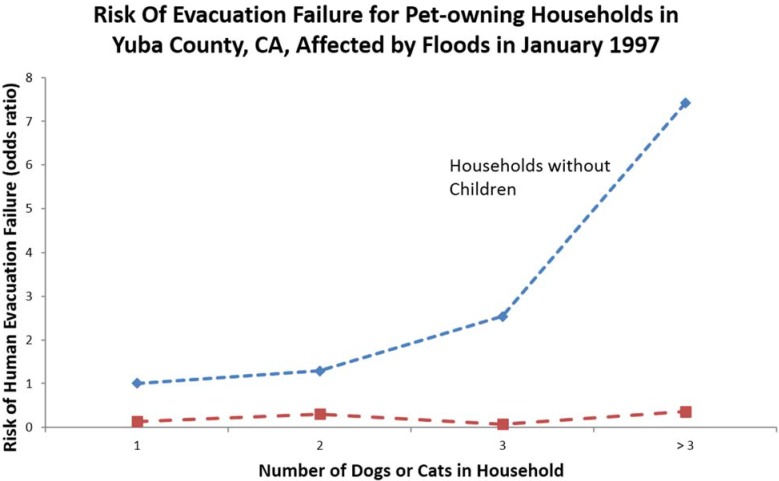
Chart correlating the likelihood of pet owners not evacuating with the number of pets owned and whether the household has children [21].

Local ordinances can also mitigate animal disasters by enacting strong animal control laws and backing this up with enforcement. For example, ordinances that implement effective spay-neuter programs for dogs and cats would reduce the number of strays in a community, which in turn likely reduces the number of stray animals that emerge in the wake of disasters. Regulations limiting the number of animals people can keep also establishes expectations for the public to limit the number of animals under their care to a reasonable span of control. Such laws create a mindset amongst animal owners that they should not own more animals than they can take care of, especially considering the potential consequences of disasters. Studies have shown a direct correlation between the number of animals in a household and the chance of those pet owners not evacuating, Figure 3 [21,22]. Furthermore, restricting the number of animals that can be housed in a household prevents the potential for animal neglect. As animal neglect is in part defined as placing animals in unsafe and dangerous environments, one can assume that when people are told to evacuate during a disaster it is because the environment is unsafe and dangerous. When people house more animals than they can care for, when they leave animals behind during an evacuation, they expose these animals to unsafe environments, and therefore are subjecting the animals to neglect. When people try to provide for too many animals with limited resources they are living on the brink of disaster in which animals could suffer when, in disasters, resources that are already under limited supply become further constrained.

Although most communities in the U.S. have laws and regulations that limit the number of animals that can be housed in a residence, regrettably few communities are endowed with adequate levels of enforcement of these ordinances. Frequently, animal control agencies are subservient to various agencies, such as law enforcement or public health, which can lead to low prioritization of animal issues before, during, and after disaster strikes, because of the many other competing needs that are thought of as more directly impacting human lives. Other common deficiencies in animal control agencies are lack of trained personnel and funding for staff to maintain animal control facilities and spay-neuter programs. Chronic underfunding of animal control agencies is a deficiency in mitigation that further reduces a community’s ability to plan and prepare for emergencies and, with that, limit the effectiveness of response. Low pay of animal control workers further exacerbates a community’s inability to effectively implement all phases of emergency management, because of the resulting poor retention of trained and experienced personnel.

## 5. Response

The response phase of disasters is defined by a temporary mismatch of the need to protect life and property and the resources available to meet those needs.

The response phase of a disaster begins with the first notification of the disaster. Throughout the response there is an ongoing assessment and gap analysis to determine the requirements for resources, and these activities last until urgent needs are no longer the predominant reason for demand for resources. In practical terms the end of the response phase of a disaster should be thought of as starting when the majority of affected people are able to work regular hours again.

As with other areas of emergency management, the greatest return on investment to ameliorate the adverse consequences of disasters, such as those to animal and public health, comes from efforts in the four phases other than response. Other sections of this article exemplify the benefits and return on effort during emergency management phases other than during response. Nevertheless, the response phase is critical and a time when morbidity and mortality can be reduced in the spur of a moment, and its management is frequently scrutinized publically. Response also forces critical decisions that can set the direction communities take during the recovery phase, e.g., where choices are offered to either return to previous conditions and, therefore, retain existing vulnerabilities, or to move towards a recovery that will lead to better planning, preparedness, and mitigation for the future.

The mismatch of needs and resources gives rise to common high profile images and human interest stories, often perceived to have been brought on solely as the result of the disaster. High profile response issues that have repeatedly arisen for animals in disasters include:

- Lack of clear command and direction;
- Pet owners failing to evacuate because of their pets;
- Pet owners evacuating without their pets;
- Pet rescue of animals from premises after owners have left them behind;
- Stray animals;
- Unaccountable fundraising;
- Mismanaged volunteers.

As many of these have been recurrent issues in U.S., they reflect the effectiveness (or lack thereof) of mitigation, planning, and preparedness efforts preceding the disaster. The following provides examples of how critical issues that arise during the response could have likely been prevented during other phases of the emergency management cycle had they been given appropriate level of priority and had a comprehensive emergency management strategy been in place.

### 5.1. Lack of Clear Command and Direction

Lack of clear command and direction manifests itself as conflicting or confusing instructions to the public, e.g., on whether to evacuate with or without animals, where help is available, and information on threats and risks to people and animals alike. This type of confusion originates when there is not an established Incident Command that includes addressing the needs of animals and their owners.

Communities that have not engaged in appropriate planning or mitigation for the needs of animals and their owners in disasters often find themselves without a qualified or experienced person overseeing animal issues during the response to a disasters, and, therefore, animal issues are predictably disregarded by emergency management officials during a response. This can result in actions ranging from an inaction (a common problem with State Animal Health Officials without explicit authority over healthy pets); bad decisions, such as to kill stray animals (a common problem with unsympathetic law enforcement) or when inexperienced helpers incorrectly assess the severity of injuries; external groups taking charge and prioritizing response operations to fit their objectives rather than those of the community; and misuse of critical human and equipment resources by unqualified or inadequately trained responders. Appropriate planning should identify these potential short falls and develop a path by which to implement appropriate mitigation measures that establish adequate authority over decisions and resources.

During the response to large-scale disasters and during exercises many volunteers offer their services; this is particularly true for the care of animals [23,24]. It is not uncommon in large-scale events in the U.S. for people to travel long distances to offer help for animals. Although all of the volunteers are willing to help, history has shown that many volunteers who lack credentials or experience can hinder response operations and endanger human lives. Adding to that the media and much of the literature on animals in disasters reinforce stereotypical thinking of why animals become victims of disasters by focusing on the excitement of response, rather than concerning themselves with obvious underlying conflicts in the way Americans live with animals. For example, the length of time that a person owns a pet in the U.S. is considerable shorter than the pet’s life, which is an indicator that pet relinquishment is a common cultural phenomenon and representative of the reason we see recurring problems with stray animals in disasters. Although much of the literature on animals in disasters is quick to justify the *ad hoc* care of animals in disasters under the guise of a lack of commitment towards animals by emergency management personnel, this is misleading. More commonly emergency managers are looking for partners with whom they can permanently address the root causes of vulnerabilities for a community including its animals.

Although neither experience nor credentials can be acquired quickly, not having these skills should not be a reason to turn away willing help. We recommend that as part of the official preparedness and response to disasters volunteer managers with animal care backgrounds are identified who can assess volunteers’ skills and preferences and incorporate them into needed services. Directors of animal shelters, veterinary practices, and other animal care businesses are particularly suited for the role of volunteer manager, and these can be trained to become official members of the Incident Command [13].

### 5.2. Pet Owners Failing to Evacuate Because of Their Pets

Pet ownership is the single most common factor associated with human evacuation failure that can be positively affected when the threat of disaster is imminent [21,22]. Based on multiple studies of actual evacuations, 20%–30% of all human evacuation failures can be attributed to pet ownership. Research has shown that this behavior is principally a phenomenon of households without children and is directly correlated with the number of pets in a household (Figure 3). Planning and preparedness activities are key to reducing this problem. Planning efforts should identify vulnerable pet owners and preparedness efforts should develop community-based approaches to help, such as buddy systems amongst neighbors. Emergency managers can also help reduce the number of people, who do not evacuate because of pets, by offering them free cardboard cat carriers and dog leashes as officials go door to door to announce the need to evacuate with pets. Although most evacuees are able to find a place to stay on their own, prior identification of a pet-friendly shelter where owners can find temporary shelter for themselves and housing for their pets provides further encouragement to pet owners to evacuate with their pet. Transportation supplies, such as carriers, collars, and leashes can be made available on demand through agreements between local emergency managers, pet accessory retailers and other suppliers.

### 5.3. Owners Evacuating without Their Pets

**Figure 4:**
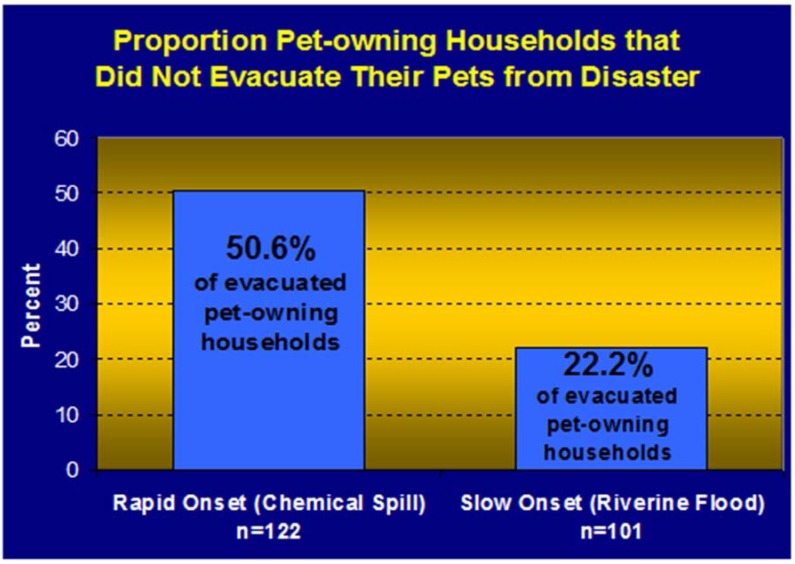
Proportion of pet-owning households that did not evacuate their pets from a slow and a rapid onset disaster [25,26].

**Figure 5:**
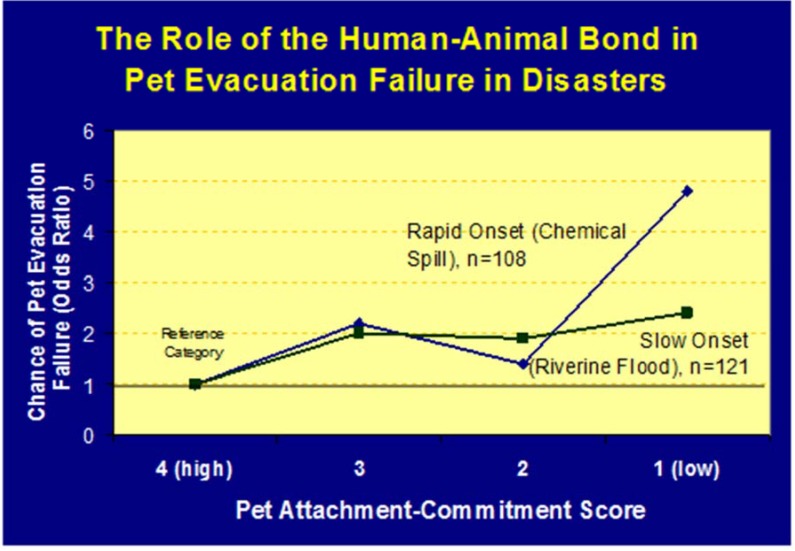
Chart showing an inverse correlation between the strength of pet owners’ bond with their pets and the likelihood that they will evacuate their pet in both slow and rapid onset disasters [28].

Pet evacuation failure (which is a form of pet abandonment), occurs when pet owners evacuate and leave their pets at home, and it has been a relatively common phenomenon in U.S. disasters (Figure 4) [25,26]. It is often erroneously attributed to inappropriate advice given by emergency management and law enforcement to leave pets behind. Although such misleading advice has been given, it has not proven to be a factor in the field or in research studies to affect pet owner’s behavior on a large scale. Research findings support that the principle reason owners leave their pets behind is because these owners have weak bonds with pets at the time of (and after) a disaster [25,26]. There are many indicators and supporting evidence for this statement. The strongest support comes from peer-reviewed research that shows that the lower an owner’s pet attachment score is the more likely they are to evacuate without their pets. Other indicators of a weak bond with pets include pets that do not have collars or visited a veterinarian in the year preceding a disaster, both of which are surrogate measures of pet attachment (Figure 5) [25,26]. Affecting this detrimental behavior for the better is difficult, but encouraging appropriate behavior is most likely to succeed through public awareness (preparedness) campaigns such as “If it’s not safe for you, it’s not safe for your animals” and public education campaigns with advice on what pet owners can do to behave appropriately in disasters—which is to evacuate with pets. *Vice versa*, looking at things from a public safety perspective, knowing that the strength of the human animal bond is a strong indicator for owners behavior in disasters, advice on the right thing to do for animals positively reinforces the human animal bond and will likely encourage appropriate evacuation behavior amongst pet owners [27].

### 5.4. Pet Rescue of Animals from Premises where Owners Have Left Them Behind

**Figure 6:**
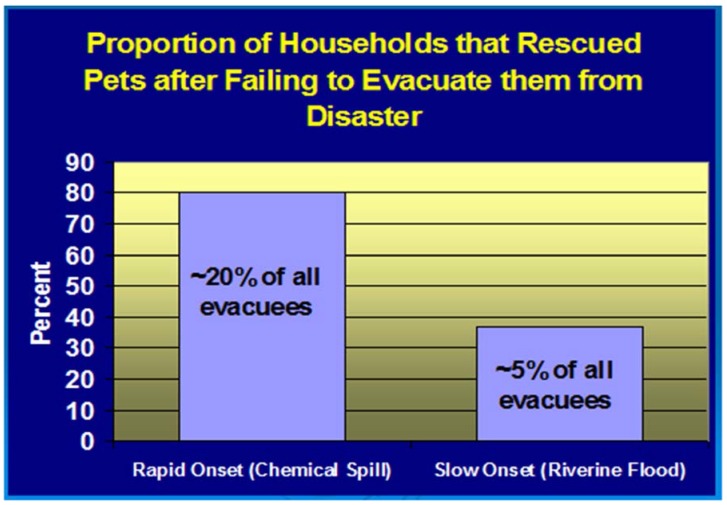
Proportion of all evacuated households that attempted to rescue pets after the owners evacuated without their pets from a slow and a rapid onset disaster [28].

Amongst the consequences of people leaving their pets behind are later attempts by owners to rescue their animals after they have evacuated. This is a rare, albeit very dangerous and often high profile behavior (Figure 6) [28]. In most cases the desire to rescue a pet is the result of peer pressure and from media stories about abandoned pets that are at risk of hazardous exposure. Less common are owners who were not at home when evacuation orders were given and, despite trying, are not given access to their homes. In either case the risk to human life should be evaluated and if deemed insignificant, animal rescues are best conducted jointly under the direct supervision of trained emergency response and animal care personnel who can determine if an animal is amenable to evacuation without delay or risk of injury to response personnel, owner or animal [24].

Pet evacuation failure and subsequent attempts at pet rescue also provide a unique opportunity for public education that role-models appropriate evacuation behavior. Based on the data from the study cited above [28] for every pet not evacuated there are at least as many that are evacuated. Officials can capitalize on this fact by making a point of rewarding appropriate behavior through public statements and media briefings that laud and illustrate responsible owners’ evacuation behavior.

### 5.5. Stray Animals

The abundance of stray animals has been a relatively common concern after major disasters in the U.S. Typically these animals are portrayed as having been tragically separated from their owners and that misconception is reinforced to the public through media stories of owners frantically searching for their lost pet. Although there are bound to be some cases that can be accurately described to have unfolded in that way, the vast majority of animals found ownerless after a disaster do not fit that mold. Studies have shown as far back as the 1970s that animals found after disasters are much more typical of the pre-existing stray animal population than pets cared for as “family members”. More recent data show that cats and dogs found after disasters are more representative of pets surrendered to humane shelters rather than house pets. For example, after Hurricane Floyd in 2008 only three out of 400 dogs entering a temporary shelter had been spayed or neutered, and a large proportion of the dogs found were pit bull types (whereas the average proportion of household pets that are pit bull types is only about 3%, the proportion of breeds that are pit bulls admitted to animal shelters is 30%–40%). Other typical characteristics of animals found after disasters are that they are older or younger (puppies and kittens) than the average family pet, have no identification, have evidence of chronic disease, and often are behaviorally maladapted. Finally, the only peer-reviewed study on the subject indicated that only about 10% of stray animals found after a disaster were reunited with their original owner and that the initiative to find a lost pet is more or less entirely the result of the owner’s efforts [29].

Further empirical evidence suggests that the number of animals found after a disaster approximates the number of stray animals one would expect to find in the affected area. Although strays are not easy to count during normal times (estimates are that approximately 15% of cats and dogs in a community are strays), they become visible when people have been evacuated, and the strays’ usual sources of food and habitation are disrupted. Further circumstantial evidence indicating that animals found after disaster are strays at the time comes from informal surveys of animal shelters in disaster-impacted areas, where it has been found that the number of stray animals admitted to the shelter in the months after a disaster decreases proportionally to the number of animals found in affected area. In other words animals found at disasters sites are mostly likely a reflection of the underlying problem of pet overpopulation in U.S. communities. During the response to disasters, effective public service announcements (PSA) can raise awareness of this issue and drum up support for effective recovery and mitigation programs for better pet population control in the affected community.

For animals and owners that have been separated as a result of the disaster everything must be done to help reunite them. Conscientious owners will do everything they can to find their pets, and they must be given every opportunity to do so, including being advised where known sightings of animals occurred, where pets are housed, and making available searchable online databases to identify and find their pets. However, part of the challenge of reuniting lost animals with their owners is lack of standardized descriptions to be able to sort through databases [30]. Whereas owners often describe miniscule details of their pet’s physique in the hope that these details will distinguish their pets, these details are often not initially helpful to responders having to screen hundreds of unfamiliar animals. Tools available through social media that convey real time information offer promising solutions to reunite lost pets with conscientious owners, but these tools are yet to prove themselves as affecting overall reunion rates.

One of the management challenges that animal shelters face in disasters is not adapting their adoption and euthanasia policies in a way that would support owners needing extra time to find and be reunited with their pets. Policies, *i.e.*, a mitigation activity, should be adopted in disasters that allow shelters to extend the waiting period for release of pets for at least an additional 3 weeks after a disaster. And, in cases where the original owner cannot be found, new owners should be required to sign a fostering agreement for the first 6–12 months of care so that, should an owner emerge later, there are no questions about who the rightful owner is. Since both of these recommendations come with potential increased resource requirements it behooves animal shelters to become fully integrated into their community’s emergency management system so that they become eligible for reimbursement for these transaction costs arising from these temporary policies.

### 5.6. Unaccountable Fundraising

Disasters create huge public empathy, and this often translates into massive donations for many causes, including the plight of animals. To be able to make the most out of donations, messages about the needs of animals have to be carefully worded. For example, after Hurricane Andrew a call for horse halters resulted in over 4 tons of halters being donated, mostly used and therefore not suitable for use; after the Murrah Building bombing in Oklahoma City, the public donated thousands of pairs of booties for the dozen or so Urban Search and Rescue (U.S.A.R.) dogs, and after Hurricane Katrina the message of desperation was so effective that people donated private planes to relocate animals (often unlawfully) all over the U.S.

As the needs for animals (housing, feed, handling equipment and environmental enrichment) that arise during a response are quickly and abundantly addressed through calls via the media, carelessly stated needs squander goodwill and potentially valuable resources. This happens when the media relies on frontline responders to describe the needs, which easily creates the impression that long-term needs do not exist or overlook underlying problems, and therefore incent the public to respond overwhelmingly with donations suited to overcome temporary inconveniences. Effective fundraising communications convey real needs, are coordinated centrally, appropriately prioritized, and given by qualified officials. Communicating through the official communications director is an example of how, through appropriate planning, tying animal issues into the official emergency management system can be highly beneficial and set the stage for a constructive recovery while the response is ongoing.

Other concerns over unaccountable fundraising range from fraud to diversion of donated funds to other causes and locations. Unaccountable fundraising can be very detrimental to long-term recovery, and in the U.S. there are many legal accounting practices that allow donated funds to be used legally for projects not supported by donors. Fundraising fraud is common in disasters [31], and raising funds for the care of animals is no exception. It is not uncommon to see new fundraising web sites pop up in the immediate wake of disasters, raise funds, and then disappear without a trace within a few weeks. To avoid misleading well-meaning donors as well as to direct donations to the affected community, communities should identify recipient organizations for sponsors before a disaster strikes and agree in advance how disaster donations will be managed. During the preparedness phase these relationships between response and donor recipient organizations can be used to promote public awareness of the designated recipient on behalf of the community as well as how to message that relationship during the response to a disaster.

Having a designated recipient organization for donated funds also provides a mechanism for proper accounting for sponsors, whose donations may be further rewarded by tax incentives. Using local resources and expertise for fundraising also reduces the potential for outside charities, which facilities and majority of staff are unaffected by the disaster, to become the major recipient of donated funds instead of the affected community. Most large charities can quickly create national awareness, web presence, and provide fundraising venues in disasters, however, few of them have actually been impacted by a disaster and as a result even fewer have developed an understanding of giving much back to affected communities.

For example, after Hurricane Katrina it was estimated that the American public donated over $40 million to help animals [32]. Although it is understandable that only a small proportion of those funds could be spent on the response, of greater concern is the evidence of how little funds were later devoted to the recovery, specifically rebuilding the animal health infrastructure of the affected areas or elsewhere in the U.S. [33,34]. Although the amount of funds raised for animals after Hurricane Katrina was extreme and probably more than the affected communities could have used to rebuild, donated funds did not appear to have enhanced response capability or investments in recovery effort to subsequent large ^-scale disasters at other locations. Many States have an Emergency Support Function (ESF) for notification of the public on what donations are needed, designated reception sites, storage and distribution of donated materials; these official ESF are also best suited to coordinate the soliciting of resources for animals.

## 6. Recovery

The recovery phase of disasters emerges while the response is subsiding. Emergency managers begin the recovery phase in the first hours of a disaster event, but the effectiveness of the early recovery planning often does not emerge until the response is subsiding. Depending on the proximity in time to the disaster, early recovery typically involves debris removal, demobilization of personnel, disposition of resources acquired during a disaster, rehabilitation of the community, preparation of After Action Reports and Corrective Action Plans. The recovery phase is the longest and most expensive phase of a disaster. Long-term recovery involves planning for a better future where the events of the past do not recur. In other words, the recovery phase offers many opportunities for the future, and the direction a community chooses to go depends on planning prior to a disaster, the integrity of After Action and Corrective Action Plans, as well as the level of interest and resources raised during the response. For communities that take animal issues seriously, the recovery phase is an ideal time to plan and start working towards a better future, such as embarking on a formalized approach to building a vibrant animal health infrastructure that thrives because the constant pressure from systemic root causes has been removed. Throughout this article we have given examples of what a viable animal health infrastructure could look like, what the root causes of vulnerability are to that infrastructure, and how these vulnerabilities can be addressed to improve a community’s animal and public health. During the recovery phase of a disaster, awareness of issues and the resolve to prevent or mitigate concerns for animals is high. Hence the recovery phase presents a unique opportunity in time to grasp the chance for improvement of the animal health infrastructure. Communities that have developed a comprehensive emergency management strategy that includes animal issues before disaster strikes will enjoy the greatest chance of recovering to a desired future.

## 7. Summary

Over the last two decades, many of the issues that surround animals in disasters have been seen repeatedly in different incidents in the U.S.. We have tried to exemplify these as well as point to their root causes. Common to many of these issues is a weak animal health infrastructure because of chronic pressure from pet overpopulation. Unless this root cause is addressed, the communities remain vulnerable to similar challenges they and others have faced in past disasters. Typical shortcomings in communities’ animal health infrastructure to deal with pet overpopulation before disaster strikes include: weak animal control services, including the lack of pragmatic ordinances and enforcement, poor understanding of the public of what animal and economic health benefits a community derives from animal ownership, and a limited appreciation of the size and diversity of the animal populations in a community. For example after major disasters, inadequate spay-neuter programs before the disaster have led to problems with stray animals at the disaster site, weak or poorly enforced animal welfare standards have led to human evacuation failure because of animal ownership and an inability to reunite stray animals with their owner; insufficient veterinary services result in lowering humane standards of care, both in makeshift animal housing operations, as well as when treating or euthanizing animals; shortages in animal care and feed supplies have typically been overcompensated with overwhelming donations from outside the affected community further burdening local resources and personnel.

A major challenge of mitigating animal issues has been that frequently the care of animals is treated as a stand-alone, response-centric issue, but, as many emergency managers have found out the hard way, in disasters the bond between people and animals can either interfere with overall response operations or can be managed to improve overall operations. Owners can become paralyzed by not knowing what to do with their animals, alternatively, by being involved in community planning, preparedness, and mitigation efforts, animal owners become empowered to move themselves and their animals out of harm’s way. There is no other factor contributing as much to human evacuation failure in disasters that is under the control of emergency management when a threat is imminent as pet ownership. Emergency managers can take advantage of the bond people have with their animals to instill appropriate behavior amongst pet owners in disasters by developing an appreciation for the contributions that animal ownership makes to the health of communities and by building on the human-animal-bond.

There are many well-developed resources for the care of animals in disasters in the U.S., however, the recurrence of some of the issues affecting animals in disasters in the U.S. speaks volumes as to the underlying vulnerability many communities still face. Whereas in the past much of the care of animals has been provided independent of official emergency management channels, the development of federal credentialing and nationally recognized standards for sheltering offer optimism that the care of animals is becoming more a mainstream function of emergency management.
